# Emerging drivers of DNA repeat expansions

**DOI:** 10.1042/BST20253067

**Published:** 2025-08-13

**Authors:** Liangzi Li, W. Shem Scott, Sergei M. Mirkin

**Affiliations:** Department of Biology, Tufts University, Medford, MA 02155, U.S.A.

**Keywords:** repeat expansion diseases, genome editing, repeat expansions, repeat contractions

## Abstract

Expansions of short tandem repeats (STRs) are the cause of a class of human hereditary disorders called repeat expansion diseases (REDs). Most REDs are neurodegenerative or neurodevelopmental diseases such as Huntington’s disease, myotonic dystrophy, fragile X syndrome, and Friedreich’s ataxia. Some common neurodegenerative diseases, including Alzheimer’s and Parkinson’s disease, have also been associated with STR expansions. Many cellular processes such as meiotic recombination, DNA replication, and mismatch repair have been shown to promote STR instability. However, STR instability is likely the result of a variety of factors, and many questions regarding this phenomenon remain to be answered. In this review, we summarize recent studies that propose DNA single-strand breaks as drivers of large-scale STR instability, in both dividing and non-dividing cells, and discuss additional evidence that supports this model. We also highlight the FANCD2- and FANCI-associated nuclease 1 protein, which was shown to be the strongest genetic modifier of several REDs.

## Introduction

Short tandem repeats (STRs), also known as microsatellites, are genomic DNA regions containing repeated 1–9-bp-long units. About 6.7% of the human genome is made of STRs or STR-derived sequences [1]. In some cases, STRs might serve biological functions such as in gene regulation [2]. At the same time, expansions of some STRs lead to a group of human hereditary disorders called repeat expansion diseases (REDs). REDs are predominantly neurodegenerative and neurodevelopmental diseases including Huntington’s disease (HD), myotonic dystrophy, fragile X syndrome, Friedreich’s ataxia, and many others (reviewed in ref. [3]). With the advances of long-read sequencing, more REDs are identified each year [4,5], such as cerebellar ataxia, neuropathy, vestibular areflexia syndrome (CANVAS), caused by (AAGGG)_exp_ in the *RFC1* gene, and late-onset cerebellar ataxias, caused by (GAA)_exp_ in the *FGF14* gene [6]. Other neurodegenerative diseases such as Parkinson’s [7,8] and Alzheimer’s disease [9] were also suggested to be associated with STR expansions. In addition, expansions of GAAA repeats were detected in ~40% of renal cell carcinoma cancers [10]. Genome-wide microsatellite instability is also characterized in various types of cancer [11]; however, the mechanism responsible for this type of instability is different from locus-specific STR instability: it is driven by defects in the mismatch repair (MMR) pathway and results in small-scale instability (reviewed in [12]). In this review, we focus on recent advances in understanding the drivers of locus-specific STR instability.

In most unaffected individuals, RED-associated alleles contain fewer than 10–30 repeat units and are stable in size. These are considered normal-size alleles. If normal alleles undergo repeat expansions, they can become premutation alleles which are exponentially more likely to expand with additions in repeat length. Individuals with premutation alleles usually do not display symptoms but are considered carriers since their offspring are very likely to inherit even longer repeat alleles that are pathogenic. It is generally believed that the majority of REDs are caused by STRs that have an ability to form non-B DNA secondary structures, such as DNA hairpins, slipped-strand DNA, triplex H-DNA, quadruplex G4-DNA and i-motifs, and DNA unwinding elements (reviewed in refs. [13–20]). In several cases, formation of these alternative DNA structures by expandable repeats has been directly demonstrated *in vivo* [21–23].

Various cellular pathways are implicated in repeat expansions. An early hypothesis was that repeat expansions could result from unequal crossing over during meiosis [24,25]. However, the lack of coinciding exchanges in flanking markers at repeat loci [26,27] suggests that meiotic crossing over is unlikely to contribute to repeat expansion in most cases. A notable exception is (GCN)_n_ repeats, expansion of which leads to polyalanine runs in proteins. These intergenerational expansions in human pedigrees are quite small in scale and likely result from the unequal crossing over [28]. Other types of recombination events such as sister chromatid exchange during mitosis should not result in changes in flanking sequence. In fact, mitotic recombination has been shown to promote repeat instability in bacterial and yeast experimental systems for studying repeat expansions [29–31].

The second major hypothesis to emerge was that expansions could occur during DNA replication, owing to strand slippage and formation of unusual DNA structures on nascent DNA strands [32]. This hypothesis was strongly supported by data showing that replication fork progression through expandable repeats was compromised in every experimental system studied from bacteria to human cells [33–39]. Furthermore, large-scale expansions appeared to depend on DNA replication in several model systems [22,40,41], as well as in patient cells [37]. It was quickly noted, however, that: (i) repeat expansions also accumulate in post-mitotic cells in patients [42–45] and (ii) while both expansions and contractions of DNA repeats occur during their replication, there is a strong bias for expansions in human pedigrees.

A third popular idea came from the mouse models. It was demonstrated that knocking down MMR genes *MSH2* and *MSH3* precluded intergenerational and somatic expansions of (CAG)_n_/(CTG)_n_ repeats [46–48]. It was further shown that mutations in other MMR proteins such as PMS1, PMS2, and MLH1 similarly preclude repeat expansions [49–52]. This led to a counterintuitive hypothesis that instead of stabilizing STRs, as was previously demonstrated for microsatellite instability in cancer (reviewed in refs. [12,53]), MMR proteins can promote repeat expansions by either stabilizing or enabling the formation of slip-stranded or other non-B DNA structures formed by expandable repeats (reviewed in refs. [12,54–56]). Supporting this idea, genome-wide association studies (GWAS) data have shown a correlation between mutations in MMR genes and delayed age-of-onset in RED patients [57,58]. Since MMR proteins are believed to preferentially stabilize slip-outs in nascent DNA strands, the MMR-driven instability hypothesis readily explains the higher levels of repeat expansions than contractions. This hypothesis can also account for the expansions observed in post-mitotic somatic cells. Consequently, it became a dominant idea in the repeat expansion field. A caveat, however, is that instability driven by MMR proteins is usually relatively small in scale, such that only a few repetitive units are added per human cell division or mouse generation [48,51,59,60]. While small-scale expansions can result in the gradual progression from normal-size alleles to premutation alleles and ultimately to pathogenic alleles, the question remains: what mechanisms are responsible for large-scale expansions adding dozens or even hundreds of repeat units at once in both dividing and non-dividing cells?

Several recent studies have implicated DNA nicks and gaps as previously uncharacterized drivers of large-scale repeat instability. A DNA nick is a break in the phosphodiester backbone of one strand; a DNA gap occurs when a segment of one strand in a duplex is missing, thereby exposing a single-stranded segment of the other strand. Here, we discuss these studies and how they could potentially provide a unifying model for repeat expansion in human pedigrees. In addition, we discuss the FANCD2- and FANCI-associated nuclease 1 (FAN1) protein, which has been identified by GWAS as the strongest genetic modifier of several REDs, its repeat-stabilizing ability in relation to the MMR pathway, and how it could be implicated in nick-mediated repeat instability.

## Nick-mediated repeat instability

The first analysis of the effect of DNA nicks on STR instability was conducted in 2016 [61]. Nicks within (CAG)_n_ repeats in human cells were induced using the CAG sequence as the PAM site for CRISPR/Cas9 nickase. Since their sgRNA was completely repetitive, Cas9 nickase generated multiple nicks within the long (CAG)_n_ run in a relatively non-specific manner. This approach led to both repeat expansions and contractions, but with a strong bias for contractions versus expansions [61]. The authors hypothesized that the nicks caused an accumulation of gaps and the formation of repetitive hairpins or loop-outs, ultimately leading to loss of DNA repeats upon DNA repair synthesis through those template structures [61]. Recently, their follow-up study found that nick-induced contractions are dependent on transcription rather than replication, and they can occur in both dividing and non-dividing cells [62]. Another study by the same group has explored the therapeutic potential of nick-induced contractions in both HD and myotonic dystrophy 1 (DM1) patient-derived neurons. Cas9 nickase with sgRNA targeting CAG repeats was indeed able to induce contractions of (CAG)_exp_ alleles without major off-target effects [63].

Surprisingly, however, in 2024, two other groups showed that targeting Cas9 nickase adjacent to the (GAA)_n_ or (G_4_C_2_)_n_ repeats promoted large-scale expansions in yeast or mouse model systems, respectively [64,65]. In yeast, nicks 5′ to the (GAA)_100_ repeat led to an over-two-orders-of-magnitude increase in repeat expansions. Notably, 5′ nicks also induced large-scale expansions of the premutation and even the long-normal repeat alleles, albeit at significantly lower rates than that for the pathogenic (GAA)_100_ repeat. Furthermore, DNA nicks also led to the accumulation of repetitive tracts that were several times longer than the original repeat size [64].

In mouse embryonic stem cells with (G_4_C_2_)_96_ repeats, 5′ nicks induced both large-scale expansions and contractions, with a slight bias for expansions [65]. In addition, the authors induced nicks in one-cell embryos of a mouse model with knock-in *C9orf72* (G_4_C_2_)_96_ repeats and also observed large-scale repeat instability in tissue samples [65].

**Figure 1:**
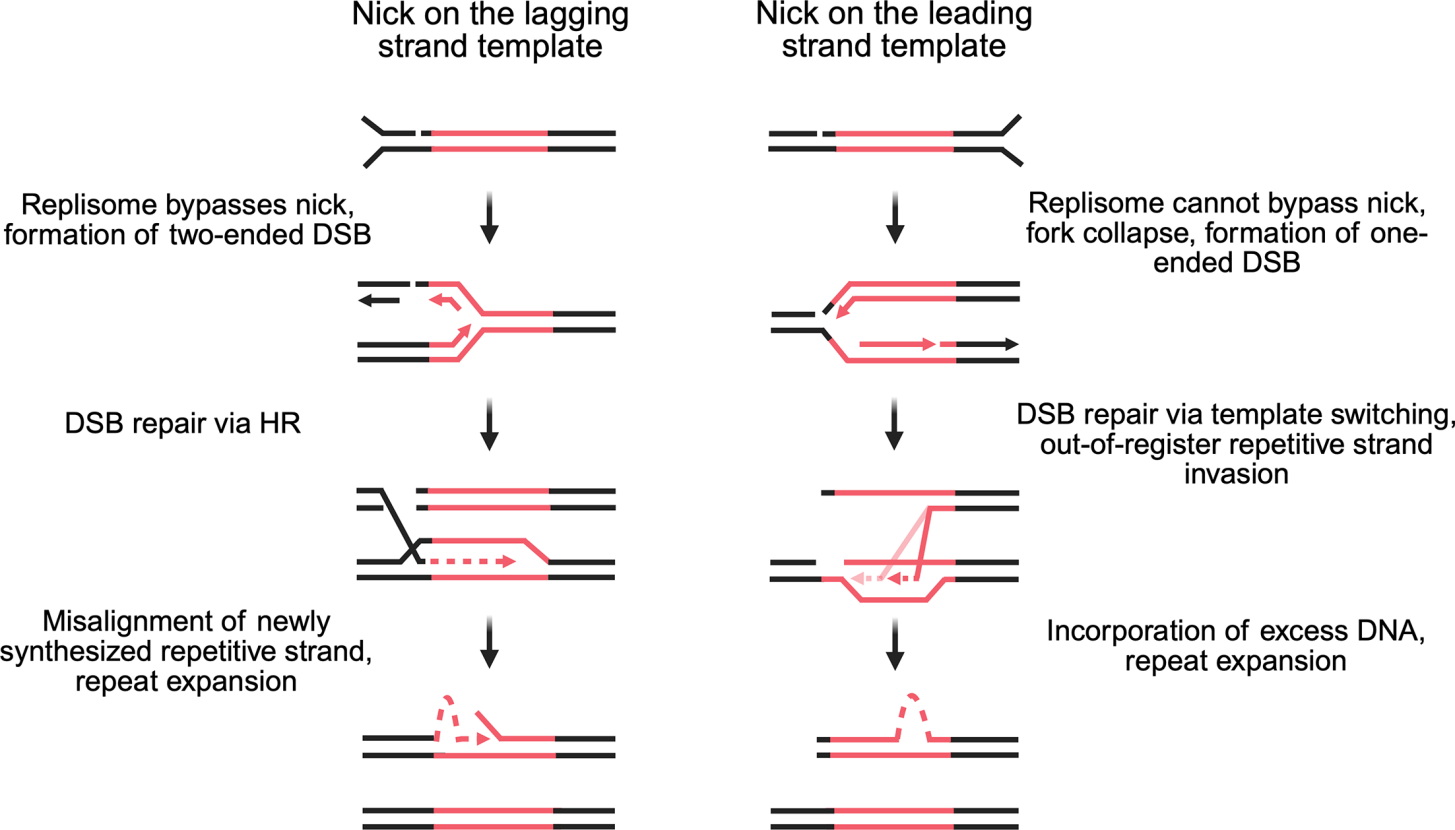
Proposed models of replication-dependent nick-mediated repeat expansions. During DNA replication, if the replisome runs into a DNA nick adjacent to the repeat tract on the lagging strand template (left), it could bypass the nick and leave behind a two-ended double-strand break (DSB). This DSB could be repaired by sister chromatin exchange; however, when the newly synthesized strand re-anneals back to the nascent lagging strand, it could be misaligned due to the nature of the repeat, eventually resulting in expansion after the next round of replication. On the other hand, if the replisome runs into a DNA nick adjacent to the repeat tract on the leading strand template (right), it cannot be bypassed and would cause fork collapse. This results in the formation of a one-ended DSB. This DSB could be repaired by template switching (TS); however, when the nascent leading strand invades the sister chromatid, it could misalign and copy excess repeat sequence, which could ultimately lead to repeat expansions. HR, homologous recombination.

These two studies proposed a model for nick-mediated repeat expansions implicating DNA replication. In brief, when the replisome runs into a nick at a repeat, it produces a double-strand break (DSB), which is then repaired via some recombination process. This recombinational replication fork repair may ultimately result in repeat expansions owing to misalignment of repetitive DNA strands in the process (Figure 1).

In line with this model, several recent studies in yeast [66,67] and mammalian cells [68,69] described mechanisms of nick repair in the context of DNA replication. Nicks induced by either Cas9 nickase or Flp recombinase are converted into one-ended or two-ended DSBs during replication, depending on their position in the replisome (reviewed in ref. [70]). Nicks on the lagging strand template can be bypassed by the replisome, leaving a two-ended DSB behind. In rare cases, nicks on the lagging strand template are sufficient to trigger fork collapse, generating one-ended DSBs with 3′ single-stranded overhangs [71]. Nicks on the leading strand template cannot be bypassed by the replisome because the CMG helicase, which translocates along the leading strand template, will fall off [72,73], leading to fork collapse and formation of one-ended DSBs. These DSBs are then repaired by recombinational mechanisms such as canonical homologous recombination (HR) in the form of sister chromatid exchange, or break-induced replication (BIR) pathway.

These findings further support the proposed model of nick-mediated repeat expansions (Figure 1). Depending on the position of a nick relative to a repeat and replication direction, either a one-ended or a two-ended DSB is formed. Subsequently, various recombinational pathways can be used for the fork repair, which range from canonical HR to BIR or template switching (TS). The differences in the mechanisms of those fork repair pathways account for the differences in genetic controls of repeat expansions triggered by DNA nicks in leading versus lagging DNA strands [64].

These models are also supported by previous studies showing repeat expansions during fork repair via BIR [41,74]. Recently, DNA nicks were also shown to drive expansions of much longer (~2-kb-long) yeast CUP1 arrays via BIR [75].

An obvious question is: why are DNA nicks more prone to inducing expansions in some cases, while in others, they induce more contractions? There are two main considerations concerning the aforementioned studies. The first is whether the nicks are positioned adjacent to or within the repeat tract. Secondly, when the sgRNA is fully repetitive, multiple nicks likely accumulate within the repetitive tract, as opposed to a single nick produced by a non-repetitive sgRNA. It was proposed that the presence of multiple nicks within a repetitive tract during transcription leads to repeat contractions [62]. In contrast, conversion of an individual nick into a DSB during DNA replication preferably leads to repeat expansions during repair [64,65]. Future studies are needed to further explore this question.

A potential therapeutic strategy is to introduce paired DNA nicks around an expanded repeat to delete it altogether [76]. Here, the mechanism is quite different from nick-mediated repeat expansions described above. Without any need for replication to convert them into a DSB, inducing paired nicks instantly generates a DSB with 3′ ssDNA overhangs. Prime editing has also been used to shorten repeat tracts without creating a DSB [77]. A prime editor consists of a Cas9 nickase, a reverse transcriptase, and a prime editing guide RNA (pegRNA). When nicks are induced by prime editors, part of the pegRNA serves as a template for addition of 3′ flaps that contain a repeat allele with the desired length. The final repair product would be a normal-sized allele with no changes to the flanking sequence.

**Figure 2:**
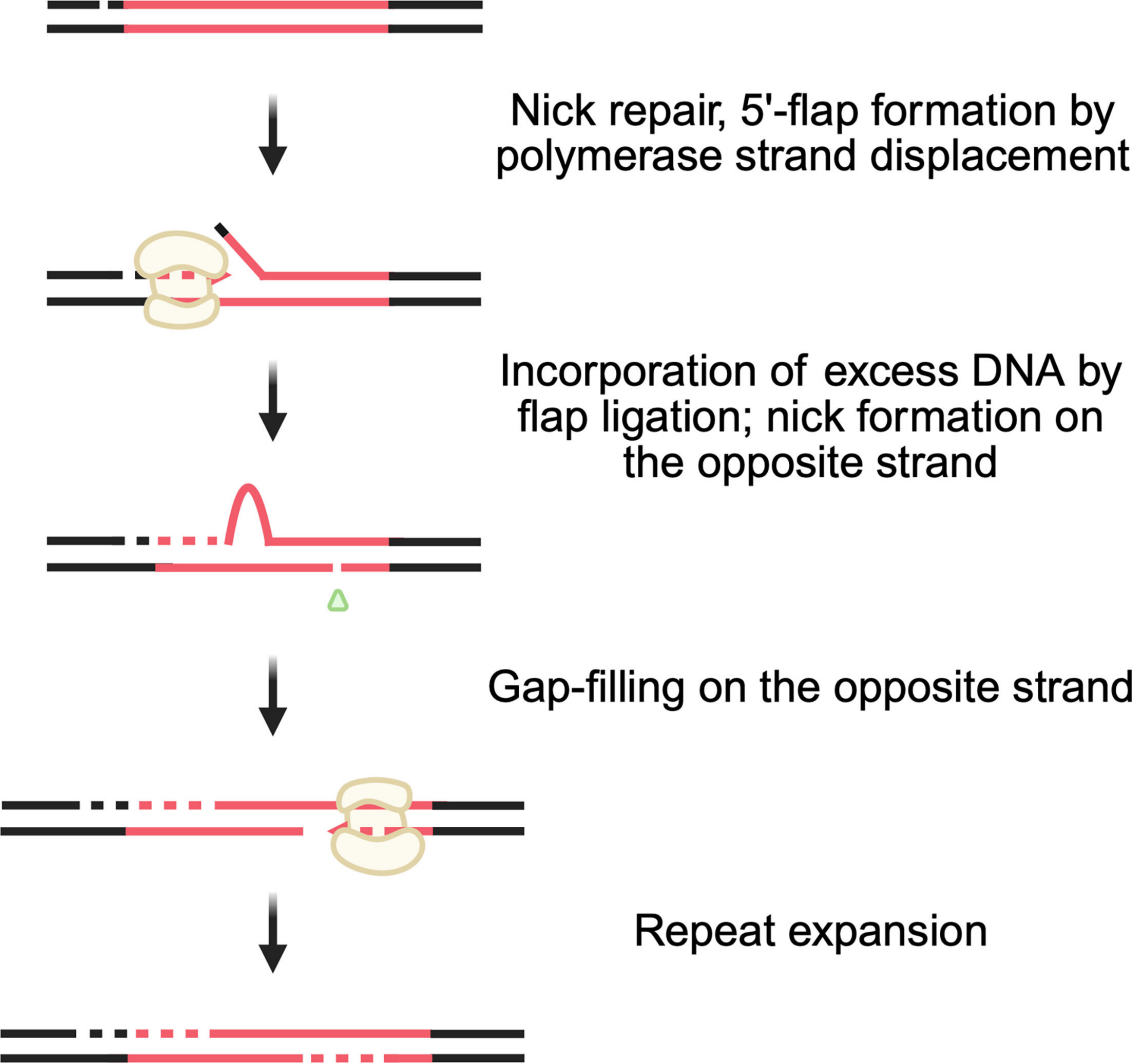
Proposed model of replication-independent nick-mediated repeat expansions. In non-dividing cells, when a DNA nick adjacent to a repeat tract is being repaired, the polymerase responsible for gap-filling, likely Polδ, could displace the 5′ end of the break, resulting in a 5′-flap. If this flap is not processed correctly and/or forms a secondary structure, it could be incorporated into the newly synthesized strand. When another single-strand break (SSB) is introduced nearby on the opposite strand, the repair process could ultimately lead to repeat expansion of both strands.

Interestingly, both studies on nick-mediated repeat expansion showed that nicks also drive expansions in non-dividing cells or somatic cells. What replication-independent mechanism could account for nick-mediated repeat expansions? During the synthesis step of nick or gap repair, DNA polymerase δ could displace the 5′ end of the broken DNA strand, forming a repetitive 5′ flap. If it is not processed quickly and/or forms non-B DNA secondary structures [38,78–80], it may subsequently be incorporated into the newly synthesized strand leading to a longer repetitive run in the repaired strand. When a second nick is introduced into the originally unbroken strand by an endogenous nuclease, its repair would ultimately result in expanded repeat tracts in both DNA strands (Figure 2).

Also, we cannot completely exclude a recombination-based mechanism of nick repair in somatic cells. While it is generally believed that HR is suppressed in G1 or G0 (quiescence) phases (reviewed in refs. [81–84]), some data point to the occurrence of recombination events in these phases of the cell cycle [85–89]. For example, recombination of (GAA)_n_ repeats was observed in quiescent yeast cells [88]. Centromeres – another example of repetitive sequences – become fragile sites in quiescent human cells [87] or mouse cells in the G1 phase [86], and their repair requires the RAD51 protein. Transcription has been shown to promote recombination in S/G2 phase [90], as well as in post-mitotic neurons [85]. It was proposed that R-loops – three-stranded RNA/DNA structures formed upon RNA transcripts’ invasion into the DNA duplex [91] – are at the heart of this process [85,90]. There are several ways that transcription can cause the formation of DNA breaks: (i) cleavage of ssDNA in R-loops [92], (ii) incomplete cleavage-rejoining reaction by DNA topoisomerases^1^ [93], and (iii) direct cleavage of the template strand by RNA polymerase, which was recently demonstrated in *E. coli* [94], and could also be true for eukaryotic RNA polymerases. Nicks or gaps can also lead to recombination without being converted into DSBs (reviewed in ref. [95]). While these ideas are thought-provoking, more studies are needed to unravel the mechanisms of nick-mediated repeat expansions in non-dividing cells.

## Gap-mediated instability

Owing to replication stress caused by the difficulties of replication fork progression through structure-prone STRs, single-stranded gaps can readily accumulate at these regions of the genome. Repair of these gaps in S-phase has been shown to drive genomic instability via recombination or TS mechanisms [96–98]. If those gaps persist into the G2 phase, repeat instability could arise from imprecise fill-in DNA repair [99–101]. If gaps remain unrepaired during the M phase, chromosome breakage, cell cycle arrest, or even cell death may occur [99,102,103].

To study the effect of gap filling on repeat instability, a (CAG)_n_/(CTG)_n_ repeat tract was integrated into a yeast chromosomal cassette that was designed to repair DSBs via single-strand annealing, an HR pathway that requires extensive resection [101]. After induction of a DSB, resection would extend past the repeat tract exposing homology for repair and generating an intermediate with either (CAG)_n_ or (CTG)_n_ serving as the single-stranded template during the gap-filling step. The authors found that the repair outcome, especially repeat instability, was dependent on the orientation of the repeat. When (CTG)_n_, which forms more stable hairpins, served as the template, gap filling led to large-scale contractions, as well as to an increase in fragility and decrease in viability. However, when the (CAG)_n_ was in the template, only expansions were observed, albeit relatively small-scaled. These findings highlight the critical role of structure formation in repeat instability during gap repair. When hairpins form within a gap, DNA synthesis can skip over repeats, resulting in repeat contraction.

Excessive accumulation of DNA nicks and gaps can occur in normal cells when the activity of proteins implicated in DNA replication and repair is inhibited. One example is the ssDNA-binding protein RPA, whose normal function is to protect single-stranded DNA gaps from the formation of secondary structures. It was shown that insufficient activity of RPA leads to massive repeat instability [104–108].

Another example is the Mcm10 protein, which is responsible for co-ordination of leading and lagging strand synthesis during replication elongation. The *mcm10-1* mutation, which destabilizes the Mcm10 protein, leads to both large-scale expansions and contractions of (GAA)_n_ repeats by exaggerating fork stalling and gap formation at the repeat [99]. Notably, the rate of expansions in the *mcm10-1* mutant was elevated much higher than the rate of contractions. This hyper-expansion phenotype was rescued by an overexpression of RPA [99], while being exaggerated in the HR mutants.

Finally, the loss of the checkpoint activator Rad9 (53BP1 ortholog) decreased (CTG)_70_ contractions due to faster resection and gap filling via earlier loading of RPA [101]. At the same time, unruly Rad9 checkpoint activation in mutants with deficient telomere maintenance, such as *cdc13-1*, promoted large-scale (GAA)_100_ repeat expansions in the course of gap repair [100].

## An emerging model for repeat expansions

**Figure 3:**
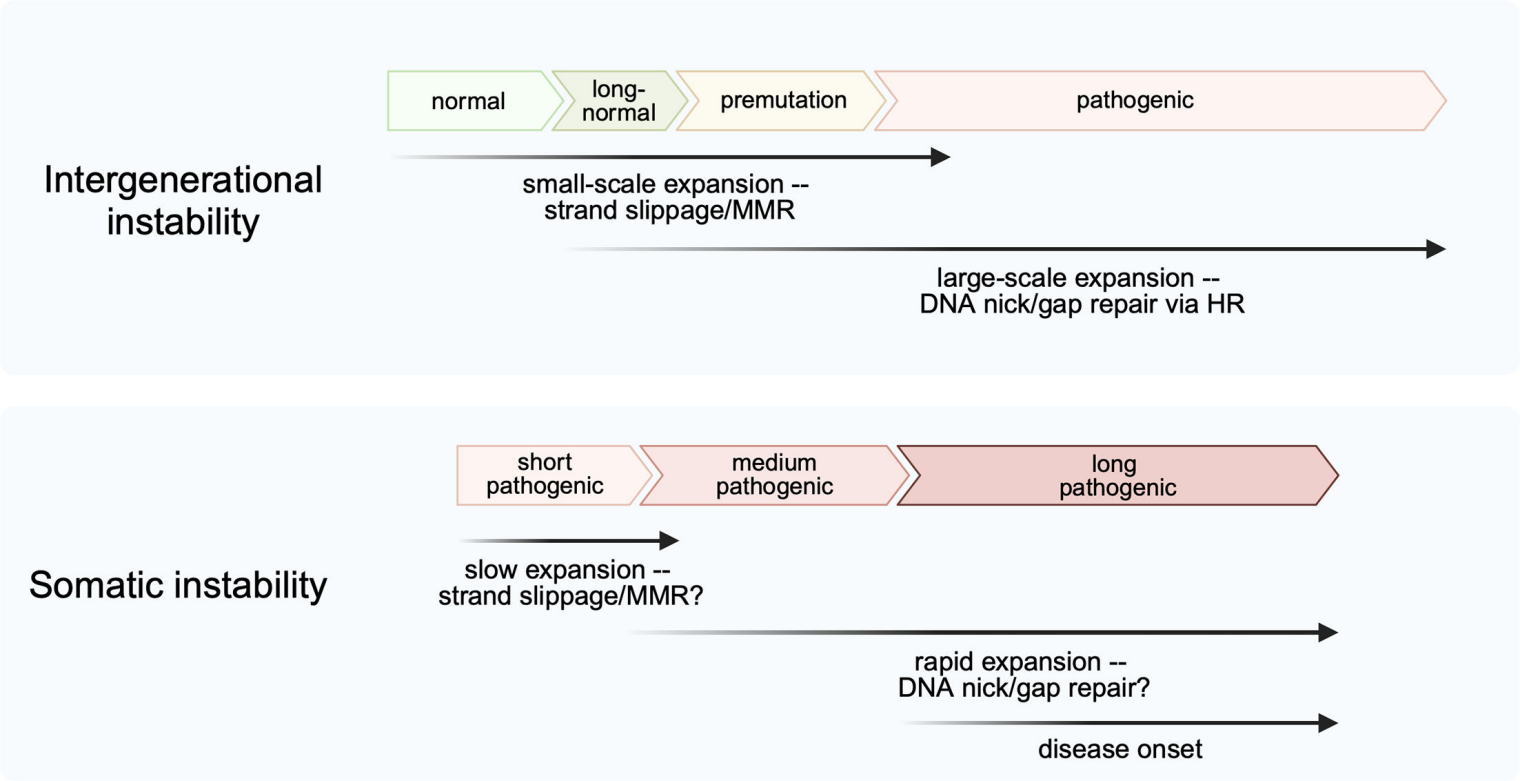
Emerging mechanisms of repeat expansions during intergenerational and somatic instability. Top: Proposed mechanisms for intergenerational instability of alleles of different lengths. For normal to long-normal alleles, MMR and strand slippage are likely the main mechanisms of expansions. These expansions are relatively small in scale, usually just a few repeat units added with each expansion event. For premutation to pathogenic alleles, expansion rates become exponentially higher and expansion scales becomes much larger. These large-scale expansions are likely promoted by DNA nick or gap repair. Long-normal alleles close to the premutation threshold could also occasionally undergo large-scale expansions. MMR and strand slippage could still promote small-scale expansions in premutation and pathogenic alleles; however, they are unlikely the main drivers of expansions. Bottom: Proposed mechanisms of expansions and disease onset in somatic cells of HD patients [45]. Inherited short pathogenic alleles slowly undergo small-scale expansions, likely via strand slippage or MMR. Over decades, they eventually become long enough (>80 repeats) for rapid expansions to take place. These expansions could potentially be mediated by nick or gap repair. Ultimately, repeat tracts exceed the threshold (>150 repeats) for abnormal gene expression and resulting in neuronal function loss and disease onset.

Altogether, the data discussed above led us to propose an emerging model for repeat expansions in human pedigrees, in which both MMR and nick/gap repair contribute to the lengthening of repeat tracts (Figure 3, top). We hypothesize that slip-outs occurring by chance in nascent DNA strands during replication or repair could lead to small-scale expansions of normal-size repetitive alleles, which ultimately brings them to the premutation size over time [109]. MMR can exaggerate this type of instability via MutS*β* binding to loop-outs or non-B structures formed by repeat sequences, MutL*γ* cleavage of the structure, and subsequent strand misalignment and gap filling [110]. Alternatively, for long-normal repetitive alleles close to the premutation length threshold, nick or gap repair could lead to large-scale repeat expansions.

Once the length of a repeat tract reaches the premutation range, its subsequent large-scale expansions driven by DNA nick/gap repair could swiftly convert it into a pathogenic allele. The resultant pathogenic alleles are even more susceptible to further large-scale expansions since longer repeat tracts can accumulate more DNA damage, thereby hastening age-of-onset and worsening disease progression. In this scenario, MMR can drive additional repeat instability, further contributing to disease progression.

As for somatic cells, a somewhat similar model was recently proposed for HD disease progression [45]. It was shown that inherited shorter pathogenic alleles are not sufficient to cause any defect in *HTT* expression, but rather somatic expansions that result in alleles with more than 150 CAG repeats are responsible for the late-onset deterioration of the neuronal functions [45]. Consequently, the authors proposed that there are two stages of somatic expansions (Figure 3, bottom): decades of small-scale changes, possibly dependent on MMR, that slowly expand shorter pathogenic alleles until they reach a critical length threshold, upon which large-scale expansions quickly accumulate resulting in disease onset.

## Sources of single-strand DNA breaks

Single-strand DNA breaks can arise from both stochastic damage and/or during normal or disrupted cellular and molecular processes (reviewed in ref. [111]). DNA-damaging agents such as reactive oxygen species (ROS), UV, or ionizing radiation could directly induce single-strand breaks (SSBs) by breaking the sugar-phosphate backbone or indirectly by triggering DNA repair pathways. For many DNA repair pathways including MMR, base excision repair (BER), nucleotide excision repair, and ribonucleotide excision repair (RER), the lesion or loop-out usually needs to be excised by nucleases, resulting in the formation of a nick or a small gap [112–114]. These steps are normally followed by DNA synthesis or gap filling and are considered programmed breaks. However, if they are somehow disrupted, the nick or gap could persist and lead to repeat instability via mechanisms discussed above.

MMR is one of the most studied mechanisms of repeat instability in the context of various REDs, which implicates MutL*γ* nicking of a DNA strand adjacent to a non-B DNA structure bound by MutS*β*. Supporting these ideas, it was recently demonstrated that knockdown of a ZNF850 protein, which binds to CTG repeats and co-localizes with MSH2/MSH3, significantly stabilized the repeat in DM1 patient-derived induced pluripotent stem cells [115]. This suggests that ZNF850 promotes CTG repeat instability by recognizing and binding to repeats and recruiting MutS*β*, which has been associated with repeat instability.

In addition, it was demonstrated that a knockout of the *OGG1* gene encoding 7,8-dihydro-8-oxoguanine-DNA glycosylase precluded somatic repeat expansions in HD transgenic mice, pointing to the role of BER in repeat instability. The so-called ‘toxic oxidation cycle’ was proposed based on this result [116]. It postulates that removal of oxidated bases by DNA glycosylases followed by APE1 cleavage creates DNA nicks or small gaps. During DNA nick repair, the repetitive 5′-flap could adopt a non-B DNA structure, which ultimately leads to repeat expansion upon flap ligation. Expanded repeat tracts have an even higher risk for oxidative damage, thereby repeating the toxic cycle. Supporting this idea, neurons are shown to have increased levels of BER activity [117]. Alternatively, unrepaired DNA nicks could be repaired by HR as described above.

There are many ways that single-strand interruptions can occur during DNA replication. First, during lagging strand synthesis, nicks or gaps are processed during Okazaki fragment maturation by RPA, flap-endonucleases, and ligases. In fact, one of the first models of repeat expansions implicated flap-endonuclease Rad27 (FEN1 in humans) [118]. Mutations in the *RAD27* gene were indeed shown to dramatically increase instability of various tandem DNA repeats [119], including expansion and contractions of trinucleotide repeats [120–124]. It was hypothesized that the ligation/equilibration of unprocessed Okazaki fragment flaps could account for repeat expansions [121,124–126], while gap accumulation leads to massive contractions [104]. Notably, however, some of the effects of Rad27 deficiency on repeat expansions were quite similar to those caused by recurrent DNA nicks, most strikingly normal-size repeats readily expanded in *RAD27* knockouts [121,122]. The interest in flap-endonuclease model was somewhat muted when it was shown that the mammalian ortholog of Rad27, FEN1, was not involved in somatic hypermutability of (CTG)_n_ repeats in a knock-in mouse model for DM1 [127]. Subsequently, however, FEN1 was associated with (CAG)_n_ repeat expansions in the striatum of HD transgenic mice [128]. Recent data on the role of another nuclease, FAN1, in repeat expansions (see below) have reinvigorated interest in the role of flap endonucleases in repeat instability.

Second, MMR and RER activities are elevated during replication to correct errors by DNA polymerases (reviewed in ref. [129]), meaning that intermediates with nicks or small gaps are heightened during S and G2 phases.

Third, topoisomerases are required to relieve supercoiling during replication, as well as transcription. However, in the case of Top1, which creates SSBs, if it cleaves at a mis-incorporated ribonucleotide, the 2′-OH of the ribose may attack the 3′-phosphotyrosyl linkage between Top1 and the ribonucleotide, which would generate a 2′,3′-cyclic phosphate end and result in an irreversible break [130].

Finally, bulky DNA lesions could trigger fork stalling and/or repriming [131], which would promote gap formation.

It is worth noting that repetitive regions have been shown to be hotspots of DNA damage [132] and recombination events [133,134]. Formation of non-B secondary structures would result in single-stranded regions that are particularly vulnerable to DNA damage [135,136]. In addition, these structures act as roadblocks during DNA replication and induce fork stalling [35,38,39,137,138], which can also lead to ssDNA gaps.

Yet another source of single-strand interruptions is the apolipoprotein B mRNA-editing enzyme, catalytic polypeptide (APOBEC) family. APOBECs are cytidine deaminases with a variety of functions important for human health and immunity [139]. However, members of the family APOBEC3A and 3B have been identified as key drivers of mutagenesis in cancer [140,141]. APOBEC3A and 3B have cytosine deaminase activity which converts cytosine on ssDNA into uracil [141,142]. Uracil would then be processed by BER which generates abasic sites and subsequently SSBs [143]. Unprocessed uracil can lead to formation of ssDNA gaps through PrimPol [144,145]. Abasic sites have also been shown to trigger fork stalling and eventually post-replicative gaps [146]. Interestingly, there is elevated APOBEC3A and 3B mutation signature at hairpin-forming sequences [147,148], likely due to the preference for the single-stranded hairpin loop as a substrate [149]. Very recently, expression of the human APOBEC3A and 3B in yeast strains with long (CAG)_n_/(CTG)_n_ tract resulted in profound repeat instability, which depended on the activity of MutL*γ* nuclease [150]. The authors proposed a model where uracil resulting from cytosine deamination by APOBEC3A or 3B at repetitive hairpins is removed by uracil DNA glycosylase Ung1 followed by the cleavage by apurinic/apyrimidinic endonuclease Apn1, thereby generating a nick. In addition, MutL*γ* could cleave either the hairpin structure or U–G mismatches, resulting in more nicks and even gaps. Repair of the resultant nicks or gaps could lead to repeat instability.

## FAN1: at the crossroad of MMR and DNA nick repair

FAN1 encodes for a structure-specific nuclease with both endo- and exonuclease activity (reviewed in ref. [151]). It was first identified as a participant in the DNA interstrand cross-link (ICL) repair via its interaction with FANCD2 [152–155]. However, it has since been shown that FAN1 acts outside of the Fanconi anemia pathway [156] and its ICL repair activity is independent of FANCD2 *in vitro* [157–159]. In addition to ICL repair, FAN1 is involved in recovery of stalled replication forks [160–163] and to a certain extent, in HR [154,155]. These functions of FAN1 are largely defined by its nuclease activities. As a structure-specific endonuclease, FAN1 can recognize branched-out structures including 5′-flaps, D-loops, and three-way junctions that resemble stalled replication forks [152–155,157,158]. FAN1 also has 5′ to 3′ exonuclease activity with a preference for dsDNA substrates containing single-stranded sites of entry such as nicks, gaps, or 3′ overhangs [153–155,157].

Multiple GWAS analyses identified FAN1 as the strongest genetic modifier of HD and several spinocerebellar ataxias that are caused by (CAG)_n_ repeat expansions [164–166]. Mutations in the *FAN1* gene were associated with earlier age of onset of a disease, while variants that result in increased expression of FAN1 were shown to delay the disease’s onset [57,165,167–169]. FAN1 nuclease activity was shown to contribute to its stabilizing effect on CAG repeats as well as CGG repeats [110,170]. However, other data imply that FAN1 nuclease activity has no effect on CAG repeat instability [171].

Although the exact role that FAN1 plays in various DNA repair pathways still remains unclear, it has been shown to interact with several MMR proteins such as MLH1, PMS2, and PMS1 [153,171–173]. FAN1–MLH1 interaction was shown to protect against CAG/CTG repeat instability [171,174], possibly by promoting error-proof DNA repair of non-B secondary structures formed by those repeats [173]. FAN1 has also been shown to share some redundancy with EXO1 in the resection step of the MMR pathway [175], and *FAN1 EXO1* double knockout has a stronger effect on promoting CGG repeat expansion than either of the single knockouts [176]. Altogether, this seems to suggest a role of FAN1 as a part of the MMR pathway.

At the same time, both knockout of *MLH1* in a mouse model [174] and knock-down of *MSH3* in a patient-derived cell line [177] rescued an increase in CAG repeat expansion caused by *FAN1* loss, which implies that FAN1 protects against MMR-dependent repeat instability. Furthermore, it was shown that FAN1 can promote CAG repeat stability by sequestering MLH1, thus inhibiting its binding to MSH3 [171]. These data indicate that instead of being a part of the canonical MMR pathway, FAN1 has the ability to interact with certain MMR proteins and modulate its activity. It is also possible that FAN1 can protect against repeat expansion independent of MMR since it can recognize and bind structures formed by repeats on its own [178,179] and even seems to compete with MutS*β* for these DNA substrates [179].

Peculiarly, while FAN1 has the ability to generate nicks and gaps, it was shown to counteract repeat expansions rather than promote expansions. This points to the importance of the context of the nick or gap. It is possible that nicks or gaps generated by FAN1 *per se* are more likely to be repaired correctly. On the other hand, FAN1 requires the presence of a pre-existing nick or gap to perform its nuclease activity [153–155,157], which includes repair of loop-out structures formed by STRs [179].

## Concluding remarks

Since the discovery of the first RED over 30 years ago, many groups have investigated the mechanisms of repeat expansion. Both *in vivo* and *in vitro* studies have demonstrated that multiple genetic mechanisms are implicated in the expansion process [180]. This complexity is partially the reason why repeat instability is not fully understood. In this review, we discuss novel mechanisms of repeat instability described in recent years, with an emphasis on the role of DNA nicks and gaps in large-scale repeat instability in both proliferating and post-mitotic cells.

Intriguingly, different studies have demonstrated that DNA nicks can bias repeats for either expansion or contraction. As discussed above, this might depend on the interactions of individual or multiple nicks at repeats with DNA replication and transcription machineries. Thus, DNA nicks are a double-edged sword, in the sense that they can either be explored as a therapeutic target or utilized as a therapeutic approach for the treatment of REDs. For example, in the former case, excessive ROS damage was implicated in the development of HD [181,182]. Likewise, ROS damage was shown to contribute to several other neurodegenerative diseases including amyotrophic lateral sclerosis, Parkinson’s and Alzheimer’s diseases [182–184], which are potentially associated with somatic repeat expansions.

There are still many unanswered questions regarding nick-mediated repeat instability. It was shown that nick-mediated expansions occur through recombination. However, it remains unclear how nicks adjacent to the repeat can also cause contractions, as was demonstrated in Kojak et al. [65]. If DNA nicks at the repeats can indeed cause both expansions and contractions, what accounts for the bias for expansions observed in human pedigrees? Finally, what could the mechanisms of nick-mediated expansions in non-dividing cells be?

**Perspectives**

- Expansions of short tandem repeats are responsible for over 70 hereditary disorders in humans, collectively termed repeat expansion diseases.
- Multiple genetic processes, including DNA replication, mismatch repair, and recombination, have been implicated in repeat expansions.
- Recently, massive repeat expansions were demonstrated to occur during DNA nick repair. The contribution of this mechanism to intergenerational and somatic repeat expansions is the subject of future investigations.

## Footnotes

1 DNA topoisomerases are enzymes that remove DNA supercoils introduced during various genetic processes by cleaving and then resealing DNA strands.

## Abbreviations

APOBEC: apolipoprotein B mRNA-editing enzyme, catalytic polypeptide
BER: base excision repair
BIR: break-induced replication
CANVAS: cerebellar ataxia, neuropathy, vestibular areflexia syndrome
DM1: myotonic dystrophy 1
DSB: double-strand break
FA: Fanconi anemia
FAN1: FANCD2 and FANCI associated nuclease 1
GWAS: genome-wide association study
HD: Huntington’s disease
HR: homologous recombination
ICL: interstrand cross-link
MMR: mismatch repair
RED: repeat expansion disease
RER: ribonucleotide excision repair
ROS: reactive oxygen species
SSB: single-strand break
STR: short tandem repeat
TS: template switching
pegRNA: prime editing guide RNA
